# Experimental Study on Tsunami Risk Reduction on Coastal Building Fronted by Sea Wall

**DOI:** 10.1155/2014/729357

**Published:** 2014-03-25

**Authors:** Sadia Rahman, Shatirah Akib, M. T. R. Khan, S. M. Shirazi

**Affiliations:** ^1^Department of Civil Engineering, Faculty of Engineering, University of Malaya, 50603 Kuala Lumpur, Malaysia; ^2^Department of Architecture, Faculty of Built Environment, University of Malaya, 50603 Kuala Lumpur, Malaysia; ^3^Institute of Environmental and Water Resources Management (IPASA), Faculty of Engineering, Universiti Teknologi Malaysia, 81310 Johor, Malaysia

## Abstract

This experimental study was conducted to idealize the efficacy of sea wall in controlling the tsunami forces on onshore structures. Different types of sea walls were placed in front of the building model. The tsunami forces and the wave heights were measured with and without the sea wall conditions. Types of sea wall, wall height, and wall positions were varied simultaneously to quantify the force reductions. Maximum of 41% forces was reduced by higher sea wall, positioned closer proximity to the model whereas this reduction was about 27% when the wall height was half of the high wall. Experimental investigations revealed that wall with adequate height and placed closer to the structures enables a satisfactory predictor of the force reduction on onshore structures. Another set of tests were performed with perforated wall placing near the building model. Less construction cost makes the provision of perforated sea wall interesting. The overall results showed that the efficacy of perforated wall is almost similar to solid wall. Hence, it can be efficiently used instead of solid wall. Moreover, overtopped water that is stuck behind the wall is readily gone back to the sea through perforations releasing additional forces on the nearby structures.

## 1. Introduction

In the last recent tsunami events, severe devastation was observed through terrific losses of life and significant damage on coastal infrastructures that reminds the world of the enormous vulnerability of tsunami incident. The damage in Indian Ocean Tsunami in 2004 [1], in Chile tsunami in 2010 [2], and in Tohoku Tsunami in 2011 [3] has renewed the public awareness about the destruction impact of tsunami hazards on their life and property. As of 2007, there was a greater chance of earthquake induced tsunami with a magnitude of 8 near Japan and that was happened in March 11, 2011. A devastating tsunami took place along Tohoku, Japan, that caused around 17 m inundation in some areas. In the US Pacific northwest zone, the probability of earthquake with magnitude of 9 is about 14% that might trigger 10 m inundation along the shore. Therefore, clear understanding of complex interaction between tsunami induced forces and impacted coastal structures is essential requirement for further protection strategy. The performance of structures during these disasters indicated the dimness in the current design code. Hence, more researches need to be conducted to understand the loading circumstances on the near shore structures based on both physical and numerical simulations. Lines of evidence from 2004 tsunami and 2011 tsunami showed that waves broke where the depth of water was equivalent to incident wave height. These waves inundate the shore as hydraulic bore with high speed. The present experiment used a dam break mechanism which could simulate tsunami like waves that propagated along the flume.

## 2. Background

Tsunamis are long oceanic waves that are mainly caused by earthquake in the ocean floor. Near origin, waves maintain larger wave length and smaller wave height. As they are transmitted towards the shore, their wave length decreases; however, wave height and speed increase. During transmission from the origin, they dissipate very little energy; as a consequence, they reach the shore with nearly unchanged tremendous energy. Field surveys in the affected areas indicated the weaknesses of existing guidelines in estimating tsunami forces on structures. Though a number of researches have been done on the formation and propagation of tsunami as well as interaction between tsunami and land structures, still there is room for improving tsunami researches. Tsunami loading impacts on onshore structures have been extensively studied by many researchers [4–17]. Cross [18] proposed formula for small-scale tsunami wave pressure considering hydraulic term as well as momentum term. Ramsden [15] considered translatory wave impacts on vertical wall rather than breaking waves. The measured results could be used to resolve sliding and overturning failures but failed to predict punching type's failure. However, Ramsden [15] formula is easily applied to engineering design as it considered bore height and water depth that are easily available from tsunami hazard maps. Fujima et al. [19] included both hydrostatic and inertia term in estimating tsunami forces taking into account the distance of structures from the shore and maximum inundation depth. Thusyanthan and Madabhushi [16] investigated the impact load on special types of coastal houses. Arikawa [4] also showed the effects of impact loading on vertical wall by large-scale laboratory experiments. Arnason [20] studied the interaction between different sized land structures and a hydraulic bore on dry bed. Though most of the design manuals for tsunami risk regions consider hydrostatic formulas (e.g., [21–26]), there is yet scope to improve the tsunami formulas for overland flow.

It was found in 2004 tsunami that there was worthy interaction between low-lying sea wall and hydraulic bore. The correlation between sea wall and bore subsequently affected tsunami flow that hit the following buildings. Field surveys on Phuket, Thailand, after 2004 tsunami agreed well that even small sea wall could perform well as protection measure during tsunami [27, 28]. Sea wall with adequate height could sufficiently reduce the damage level to the building fronted by the wall when compared with no wall situation. Posttsunami field survey (2004 tsunami) indicated two situations when sea wall did not provide adequate protection [27]. One is sea wall with inland slope and another one is sea wall made of sand bag core. Oshnack [29] studied efficacy of small sea wall against tsunami forces and found that small wall reflected waves significantly and decreased the tsunami forces behind the wall. Thomas and Cox [30] proposed predictive equations for reduction of tsunami forces provided by sea wall. Lukkunaprasit and Ruangrassamee [28] also found the failures of wood structures and poorly detailed reinforced structures with masonry infill by tsunami forces. This study mainly discusses the performance of sea wall in reducing the forces on the building structures placed behind the wall. Two different heights walls positioned in different locations are tested. Solid sea walls as well as perforated sea walls are used for experimental purposes. Finally, the effectiveness of placing sea wall in front of any land structures is analyzed.

## 3. Experimental Setup

**Figure 1:**
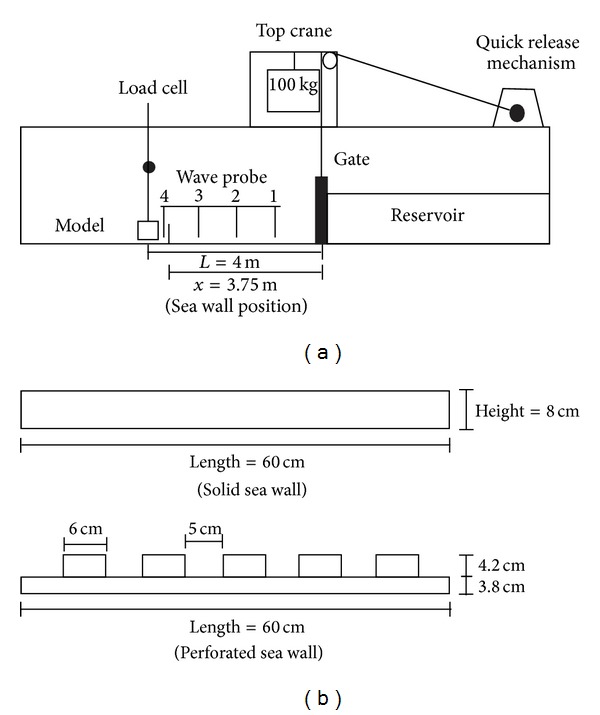
(a) Experimental setup. (b) Sea walls (solid and perforated).

Physical experiments were conducted in a wave flume of 17.5 m long, 0.60 m wide, and 0.45 m high. The flume was divided into two sections with the upstream part serving as a reservoir for generating tsunami whilst the downstream part was used to simulate tsunami propagation. The flume was also equipped with a pump to fill the reservoir and an outlet to drain the downstream part of the flume after each simulation. The experimental setup in this research was similarly used by Triatmadja and Nurhasanah [31] and Arnason [32].  Figure 1(a) shows a schematic illustration of the experimental setup. The bottom of the flume was made of stainless steel plates and, thus, the bed friction was assumed to be very small. This type of setup seems to be almost suitable for relatively flat coast that is vulnerable to tsunami attack. During simulation, models were placed at a distance of 4 m from of the dam break gate that corresponded to a distance of 140 m at a scale of 1 : 35 which represents the typical space between the shore and any onshore structure. The gate was opened very quickly and allowed water to pass along the downstream of the flume within 0.2~0.3 sec or less depending on the impoundment depth (the depth of water stored in the reservoir behind the gate). Thus, tsunami like waves were produced and transmitted as hydraulic bores towards the downstream of the flume. A wooden frame was installed on the top of the flume across the longitudinal axis of the flume. A vertical steel rod was attached to this frame. All the models were fixed with this vertical rod. At the top of this rod, there was a pin attached to the load cell. Forces were recorded as the waves hit the model that was connected with the rod. The diameter of the rod was chosen in such a way that it does not affect the recorded forces. In order to measure the water levels at various locations throughout the experiment, a series of wave probes were installed at selected stations (Figure 1(a)). The distance between the model and nearby wave probe was 0.15 m, distances between wave probes were about 0.95 m, and the wave probe at station 1 was 1 meter distance from the gate.

Building models were simulated using solid box made up of plywood (length, width, height: 8 cm × 8 cm × 8 cm) with a scale of 1/35. Researches were performed to quantify the efficacy of different types of sea wall in reducing forces on building model. The impoundment depths were selected on the basis of the maximum inundation height that would occur in a flow without the presence of the model measured at the location of the model. In this experiment, this maximum inundation height near the model structures was referred to as wave height. The sea walls used in this experiment were made up of plywood with two different heights (wall 1, H1 = 4 cm high, and wall 2, H2 = 8 cm high). The length and width for sea wall models were 60 cm and 3.8 cm, respectively. These walls were placed in front of building model on four different positions (Table 1). Subsequent overtopping was observed as the wall height was smaller than the maximum run up heights for some of the cases. At a scale of 1 : 35, the wall heights (4 cm and 8 cm) became 1.4 m and 2.8 m, respectively, which represent real wall heights more common to any coastal area. The coordinate system used in these experiments was *x* = 0 m from the gate and positive onshore in the direction of flow. The wall positions were chosen over the distance 2 < *x* < 3.75 m from the gate. The dimensionless *x*/*L* was measured to discover the appropriate location that showed the better wall performance as a protection measure. With the above arrangements, all of the waves were observed as broken and advanced along the downstream of the flume. The bore height was taken as the height of the instant water level in the inundation area. The sampling duration was selected as 90 sec, so that full inundation phases as well as any return flow could be captured conveniently. Four different reservoir depths were considered that produced different flow depths near the model. Finally, the results obtained from using higher solid wall (wall 2 = 8 cm, high) were compared with modified configuration wall containing 26% perforations (Figure 1(b)). These experiments were done to identify the effectiveness of using perorated wall relative to solid wall in force reduction as construction of perforated wall is less expensive than solid wall. All of the tests were repeated for at least three times to avoid any misleading data.

## 4. Results and Discussion

**Figure 2:**
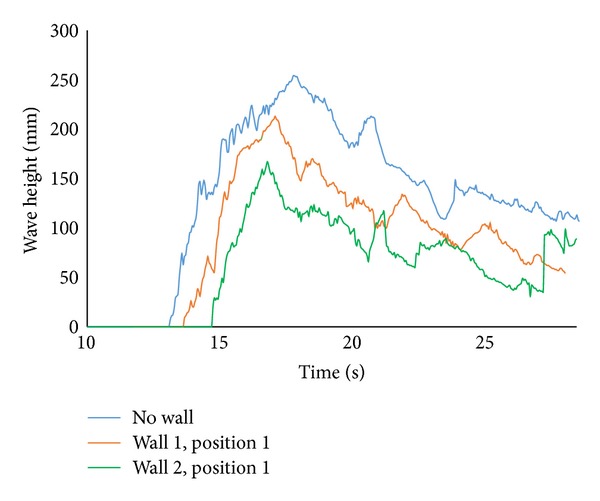
Time series of wave height for wall 1 and wall 2 in position 1 (*x* = 3.75 m from the gate).

The experiments were performed to quantify the reductions of forces on a solid building fronted by a small sea wall. Sea wall height, wall position, sea wall configuration, and wave heights were varied throughout the experimental period. Tsunami like wave was produced from dam break mechanism which propagated as a bore inside the flume [33]. An experimental time series data set measured at wave probe 4 (W4) with walls in position 1 (*x* = 3.75 m from the gate) considering 30 cm impoundment depth was presented in Figure 2. Waves were transmitted over the sea wall (orange wall 1 and green wall 2) and data were recorded by the wave probe. From this figure, it was found that as the sea wall height is increased, the wave height near the building model decreases. Other experiments were performed by placing these walls in position 2 (*x* = 3.50 m from the gate). The decreasing trends of wave height corresponding to wall height were similar to Figure 2; however, the wave height reduction rates for both the walls were smaller relative to position 1.

**Figure 3:**
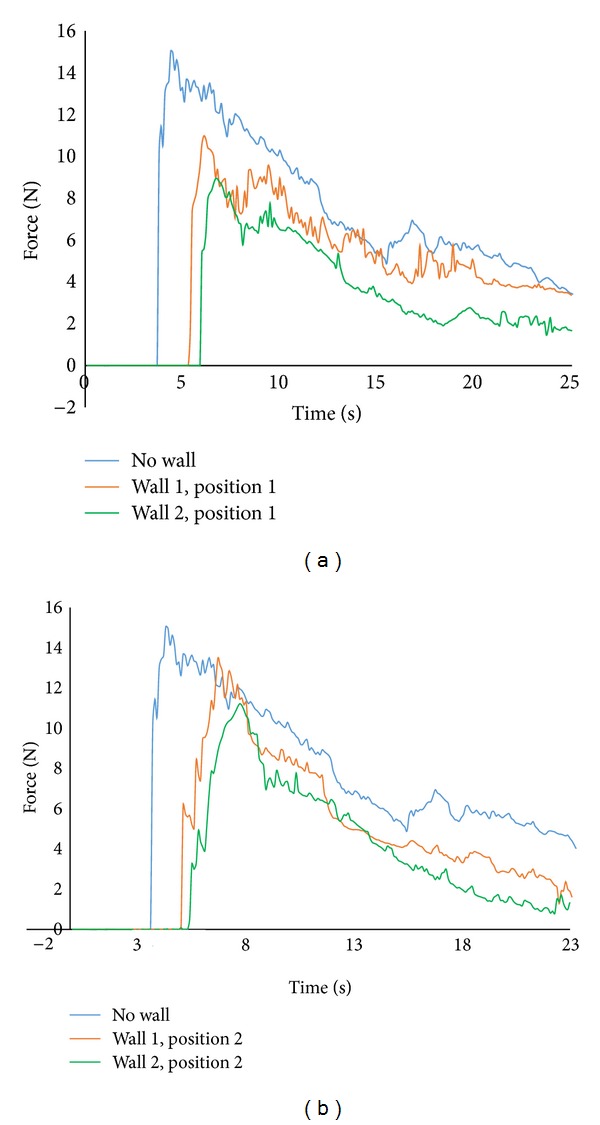
Force time history with sea wall 1 and sea wall 2 placed on positions 1 and 2, respectively.

**Figure 4:**
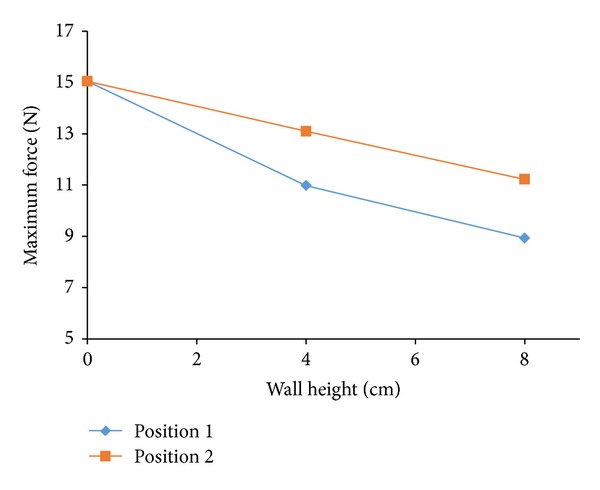
Changes of maximum forces with sea wall height.

Figure 3 shows the horizontal force time histories measured by load cell attached to the building model at an impoundment depth of 30 cm. The graph shows that there is a sharp rise of force resulting from the initial impact of bore front followed by a quasisteady force that agreed well with previous researches [19, 29, 30]. These initial impact forces (surge force) were higher than hydrodynamic forces for both no wall and sea wall conditions at higher reservoir depth. Steeper slope of the bore front is mainly responsible for this higher value as exerted forces are highly dependable on the front slope [34]. With decreasing reservoir depths, hydrodynamic force can overshoot the initial impact forces for all of the cases. Arnason [20] observed that initial impact forces exceeded the hydrodynamic forces for smaller wave heights for the case of square structure. The effectiveness of using sea wall was also presented by Figure 3. In general, results show that the presence of sea wall reduced the forces significantly on the model. Nonetheless, wall height is an important parameter in computing force reductions. For both positions, with increasing sea wall height forces are decreased; however, the reduction rate is more apparent for position 1 (sea wall that was placed closer to the building model) than that of position 2. Force profile shows that maximum force for wall 2 in position 1 was about 59% of the total force measured with no wall condition. Thus, 41% of the total forces were declined by wall 2. The reduction of forces for wall 1 shows the similar trend as that of wall 2 whereas maximum reduction is about 27%. In addition, Figure 3(a) shows that wall 1 illustrates sharp peak forces while this was not significant for wall 2. The results reveal that the higher the sea wall height, the lower the forces on the model as well (Figure 4). Besides force reduction, sea wall also caused reflection of incoming bore over the tested wave conditions. The more the height of the sea wall, the higher the refection of waves. Though wave height as well as forces was reduced in the presence of sea wall, the reduction is not proportional between them. The changes of forces with sea wall relative to no wall conditions are more apparent than those of reductions of wave height near building structure comparable to no wall condition. Therefore it could be said that wave height near to a structure fronted by a sea wall could not be a good predictor of tsunami forces.

**Figure 5:**
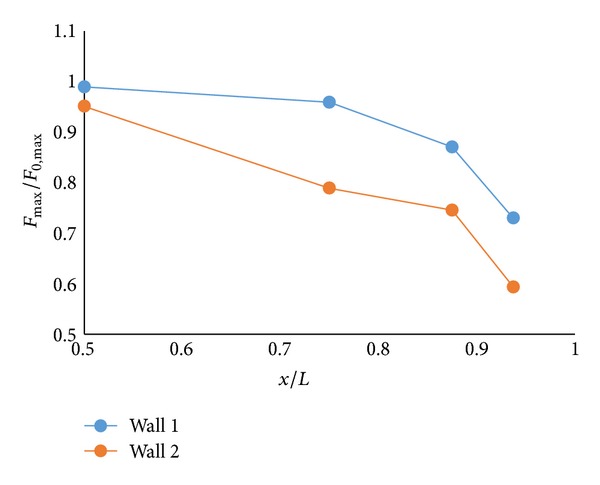
Force reduction by sea walls.

Another set of tests were performed placing wall 1 and wall 2 in position 2 (Figure 3(b)). The results show that the trends of lessening forces were almost similar as those of position 1, whereas values are smaller than those of position 1. These were 13% and 25% for walls 1 and 2, respectively. This indicated that sea wall at position 1 which was closer to the model was more effective in reducing forces. The result could be approximated clearly by Figure 5 where variations of the maximum forces with wall 1 and wall 2 relative to no wall condition were presented as a function of dimensionless ratio *x*/*L*. The graph shows that forces reduce as the wall height increases over the range 0.5 < *x*/*L* < 0.9375 m using 30 cm impoundment depths. As the walls move closer to the model, forces are decreased. Almost same trends were found with other wave conditions. This was indeed more evident with larger sea wall, that is, for the case with wall 1 (larger sea wall) where the reduction factor ranges from 0.95 (5% reduction) when the wall was located far away from the building model to 0.59 (41% reduction) when the wall was placed very close to the model. Therefore, sea wall with adequate height situated in close proximity to a structure is proved to be effective in the force reduction and could provide more protection. On the other hand, if the wall height is small and placed much closer to the structure, there might be detrimental effects on the upper floor of the structure due to localized force and large reflection. This study requires further investigation.

**Figure 6:**
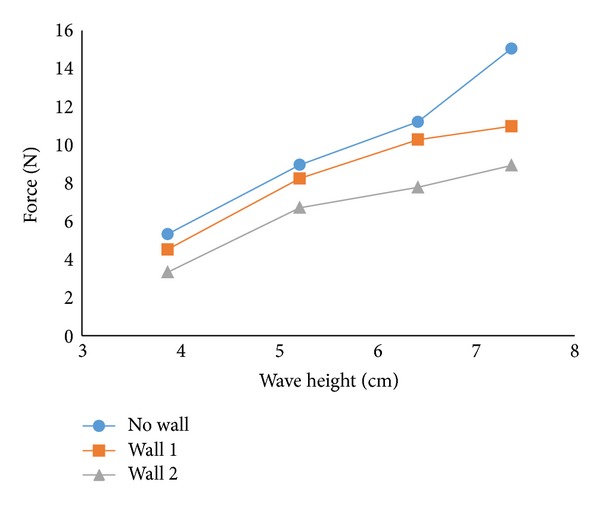
Maximum force as a function of wave height.

Figure 6 shows the changes of forces with respect to a variety of wave heights. As expected, forces are increased due to increase in wave height. This escalation of forces was more significant in no wall condition than that of conditions with wall 1 and wall 2. Higher sea wall subsequently reduced the forces over all of the tested wave height conditions.

**Figure 7:**
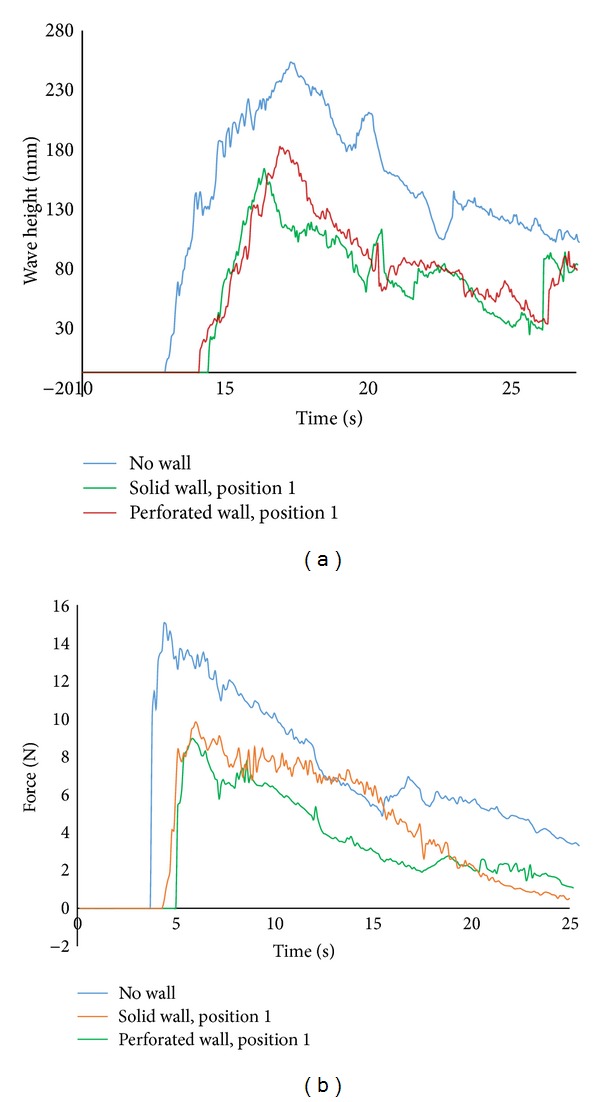
(a) Comparison of wave height time history in position 1. (b) Force time history with solid and perforated sea wall compared with no wall condition.

Another set of experiments are performed with perforated sea wall placed in position 1 (*x* = 3.75 m from the gate). The results from this arrangement are compared to no wall condition and wall 1 condition. In comparison to solid wall, perforated wall is inexpensive to construct. Moreover, when overtopping wave occurs, receding water cannot directly go back to the sea and remains stuck behind the solid wall that creates additional forces to any structure located nearby. In that case, perforated wall performs well as it could allow significant amount of water to recede through perforations. This study included the verification of the efficiency of perforated wall in the force reduction relative to the solid wall. The contribution of the perforated wall was almost similar to the solid wall and hence perforated wall could replace the solid one successfully. Wave heights along with force time histories are considered for comparison purposes.  Figure 7(a) shows the comparison of the wave height time histories between two different configuration walls (solid and perforated) relative to no wall condition. The trend of changes in wave height with time is somewhat similar between solid wall and perforated wall condition with few exceptions. In both cases, the measured wave heights are smaller than no wall condition. This indicated that the presence of sea wall decreases the wave height monotonically whereas the reduction rate of perforated wall was as satisfactory as solid wall. The trend in the variations of force time histories with solid and perforated sea wall is presented in Figure 7(b). In general, solid and perforated sea wall provides subsequent protection compared to no wall condition. The graph shows that perforated wall does not show any sharp rise in force time history as that of solid wall (both are placed in position 1) and the initial impact force is more than hydrodynamic forces. The maximum force with perforated wall is 65% of total forces. Hence, 35% force reduction is achieved when perforated sea wall was placed in position 1 instead of solid wall which provides 41% of the force reduction. Comparing the amount of force reduction, perforated wall provides nearly similar protection as that of solid wall over the tested wave conditions at position 1. Thus, it could be stated that, for this specific test conditions, perforated wall could be proved to be as successful as solid wall. Perforated sea wall is preferred to be used in lieu of solid wall owing to cost effectiveness. As the performance of both walls is quite satisfactory, lower construction cost enables the researchers to develop perforated wall rather than solid wall as tsunami protection structures.

## 5. Conclusion

This study was performed to identify the possible protection achieved by low-lying sea wall from tsunami devastation. Building structure fronted by small sea wall showed better performance than that of building without any sea wall. Types of sea wall, wall height, and wall position were varied systematically to estimate the amount of reductions in forces. The results were compared with no wall condition to verify the level of reduction. Sea walls could significantly reduce the forces on onshore structures located behind the sea wall over the tested wave conditions. Sea wall 2 (8 cm high) was perceived to be more effective in force reduction than that of wall 1 (4 cm high). For 30 cm impoundment depth, 41% and 27% force reductions were achieved by wall 2 and wall 1, respectively, in position 1, whereas these reductions were somewhat smaller in position 2 for both walls. Thus, sea walls located at adequate distance from the building structures will provide good protection against tsunami loading. Other researches were performed to evaluate the performance of perforated sea wall in lieu of solid one. As the amount of force reduction attained by perforated sea wall was almost similar to solid sea wall, this study proposed the use of perforated wall instead of solid wall. Moreover, perforated wall allowed easy declining of water to go back to sea, while solid wall trap the coming water behind and thus, creating additional forces on the building. Additionally, less construction cost of perforated wall will make it more attractive than solid sea wall. Finally, it should be noted that the results of this study were appropriate for this particular type of setup as the distance between sea wall and building structures plays a very important role in the declination of forces.

## Figures and Tables

**Table 1:** Wall positions with corresponding impoundment depth.

Wall position	*L* (distance from gate to model) (meter)	*x* = (distance from gate) (meter)	*x*/*L*	Impoundment depth (cm)
1	4	3.75	0.9375	30, 25, 20, 15
2	4	3.5	0.875	30, 25, 20, 15
3	4	3	0.75	30, 25, 20, 15
4	4	2	0.5	30, 25, 20, 15
